# Versatility of RNA-Binding Proteins in Cancer

**DOI:** 10.1155/2012/178525

**Published:** 2012-05-14

**Authors:** Laurence Wurth

**Affiliations:** Gene Regulation Programme, Center for Genomic Regulation (CRG) and UPF, 08003 Barcelona, Spain

## Abstract

Posttranscriptional gene regulation is a rapid and efficient process to adjust the proteome of a cell to a changing environment. RNA-binding proteins (RBPs) are the master regulators of mRNA processing and translation and are often aberrantly expressed in cancer. In addition to well-studied transcription factors, RBPs are emerging as fundamental players in tumor development. RBPs and their mRNA targets form a complex network that plays a crucial role in tumorigenesis. This paper describes mechanisms by which RBPs influence the expression of well-known oncogenes, focusing on precise examples that illustrate the versatility of RBPs in posttranscriptional control of cancer development. RBPs appeared very early in evolution, and new RNA-binding domains and combinations of them were generated in more complex organisms. The identification of RBPs, their mRNA targets, and their mechanism of action have provided novel potential targets for cancer therapy.

## 1. Introduction

Traditionally, it has been well accepted that cancer development is dictated in part by aberrant transcriptional events and signaling pathways. More recently, it has become clear that posttranscriptional regulation of gene expression also controls cell proliferation, differentiation, invasion, metastasis, apoptosis, and angiogenesis which influence initiation and progression of cancer [1–4]. Regulation of already transcribed messenger RNAs (mRNAs) is an efficient and rapid way to alter gene expression and plays a crucial role in tumorigenesis.

After transcription, nascent mRNAs undergo several processing steps including splicing, capping, 3′ end formation, surveillance, nucleocytoplasmic transport, and, for many transcripts, localization before being translated and finally degraded [5, 6]. The mRNA does not exist alone in the cell, and its metabolism is largely defined by bound RNA-binding proteins (RBPs). RBPs, which regulate all steps of RNA biogenesis, form dynamic units with the RNA, called ribonucleoprotein complexes (RNPs) [7]. Different sets of RBPs are associated to the mRNA at different time points and in different compartments, thereby regulating the fate of their target in a time- and space-dependent way. RBPs often provide a landing platform for the recruitment of additional factors and enzymes to the mRNA. RBPs are the master regulators of post-transcriptional gene expression and, thus, are expected to play important roles in cancer development [1]. Besides RBPs, the discovery of microRNAs (miRNA) was of great inspiration for the RNA field and provided a new powerful tool to regulate gene expression. miRNAs associate with RBPs to form microRNPs (miRNP) which regulate translation and RNA stability by binding to complementary sequences in target mRNAs. miRNPs have been found to regulate expression of factors implicated in tumorigenesis, but we will not discuss this mechanism here (for recent reviews see [8, 9]).

RBPs bind to specific sequences or secondary structures typically found in the untranslated regions (UTRs) but also in the open reading frame (ORF) of target mRNAs [10, 11]. UTRs in particular have offered more flexibility to evolution, as the constraints of encoding a protein product have not been imposed upon them. As a consequence, diverse and often conserved regulatory elements are present in the UTRs [12]. In the 5′UTR, ribose methylation of the cap structure as well as 5′ terminal polypyrimidine sequences or secondary structures such as internal ribosome entry sites (IRESs) control protein expression. Sequence elements in the 3′UTR regulate the stability of the mRNA, its translational efficiency and localization. Specific binding of regulatory proteins to these elements is achieved through RNA-binding domains (RBDs). More than 40 RBDs have been identified. Among them, the most prominent are the RNA recognition motif (RRM), K-homology domain (KH), double stranded RNA-binding domain (dsRBD), zinc finger, Arginine-rich domain, cold-shock domain (CSD), and the PAZ and PIWI domains [13]. An RNA-binding protein can contain combinations of different RBDs, which allow a high flexibility for interaction with different targets. RBP purification techniques followed by high throughput proteomics will hopefully allow us in the near future to identify new RNA-binding proteins as well as new RNA-binding domains. Powerful techniques like CLIP-seq (UV cross-linking and immunoprecipitation followed by high throughput sequencing) are helping to identify new RBP targets in a genome wide scale, as well as new RBP binding sites [14–16]. The list of RBPs, RBDs and their targets is far from being complete. New technology is proving helpful to unravel the complexity of post-transcriptional gene regulation.

In cancer cells, expression of numerous oncoproteins or tumor suppressors is under the control of specific RBPs. Splicing, stability, localization as well as translation of these mRNAs are highly regulated, often in a tissue-specific manner [6]. Many RBPs are aberrantly expressed in cancer cells and have thus a cancer-specific regulatory activity [1, 17, 18]. Deregulation of RBP expression in cancer may have its origin on epigenetic events or on miRNA-dependent controls, although the detailed molecular mechanisms are often obscure [19–21]. An additional layer of regulation is provided by signaling: the phosphorylation status of some RBPs is defined by signaling pathways that are deregulated in cancer, and this phosphorylation controls RBP activity and subsequently the expression of its target mRNAs [22, 23]. Signaling pathway alterations occur in different stages of tumor formation and are often correlated with tumor grade.

In this paper, we will summarize the different functions of RBPs in post-transcriptional gene regulation and the impact of aberrant regulation on tumorigenesis. In addition, we will discuss the conservation of specific RBPs across eukaryotes, which may yield hints on how diversity has been generated.

## 2. RNA-binding Proteins Implicated in Cancer Development

Post-transcriptional gene regulation implies factors which act at different levels of mRNA metabolism, including alternative splicing, localization, stability of the mRNA or cap-dependent and -independent translation. In this section I will introduce a subset of RBPs involved in cancer development which play key roles in each of the steps of RNA regulation, namely, Sam68, eIF4E, La, and HuR to illustrate the powerful RBP regulatory capacity in cancer.

### 2.1. Sam68 Regulates Alternative Splicing of Cancer-Related mRNAs

**Figure 1:**
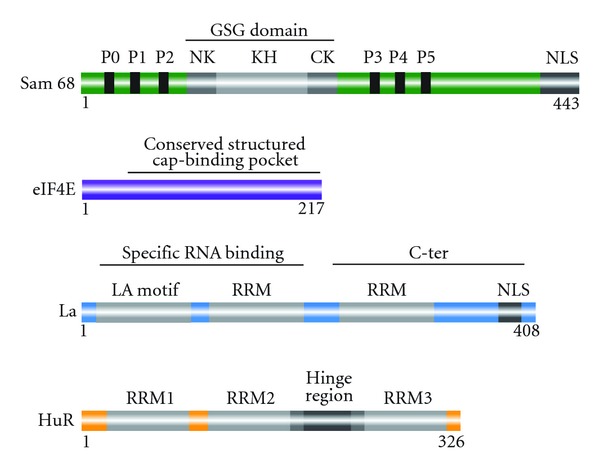
Schematic representation of the 4 RBPs discussed in this paper: Sam68, eIF4E, La, and HuR; RBDs are depicted in light gray. RRM: RNA recognition motif; GSG: GRP33/SAM68/GLD-1 domain, composed of a KH domain (KH) flanked by N-terminal (NK) and C-terminal (CK) extensions; LA: La motif. Phosphorylation sites of La are indicated in black (P0–P5). Nuclear localization signals (NLSs) are represented in dark gray. The number of amino acids of each protein is indicated.

**Figure 2:**
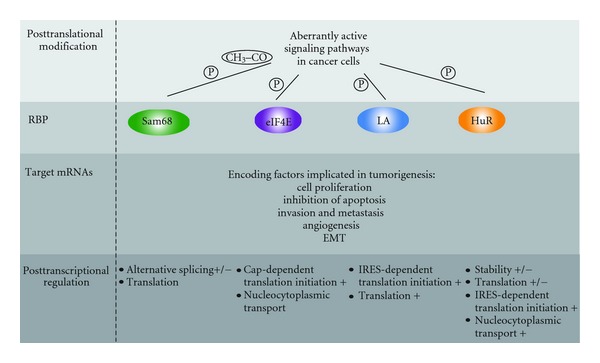
Overview of posttranscriptional gene regulation by Sam68, eIF4E, La, and HuR in tumorigenesis. In cancer cells, RBPs are posttranslationally modified by aberrantly active signaling pathways that activate their binding to targets encoding proteins implicated in tumorigenesis. The steps of mRNA metabolism regulated by RBPs are indicated. (+) and (–) specify up- or downregulation. P and CH_3_–CO indicate phosphorylation and acetylation of RBPs. EMT: epithelial to mesenchymal transition.

Sam68 belongs to the evolutionarily conserved signal transduction and activation of RNA (STAR) family of RBPs [4, 24, 25]. Sam68 is predominately nuclear but has also been detected in the cytoplasm and exerts multiple activities in gene expression, from transcription and signaling to splicing regulation [4, 26]. RNA binding is achieved by a KH domain embedded in a highly conserved region called GSG (GRP/Sam68/GLD1) domain [27] (Figure 1). RNA binding is used for splicing regulation and is modulated by posttranslational modifications, such as phosphorylation or acetylation [22, 25, 28] (Figure 2).

The role of Sam68 in alternative splicing seems directly related to its oncogenic properties. Alternative splicing (AS) allows the majority of human genes to encode for multiple protein isoforms, which often play different or even opposite roles [29]. In addition to the spliceosome, a set of RBPs are necessary to control alternative splicing [7]. Aberrant expression of RBPs in cancer can lead to deregulation of splicing, and subsequent changes in the proteome [30]. The splicing targets of Sam68 support its involvement in tumor progression [4, 31]. Furthermore, the function of Sam68 in AS is regulated by signaling pathways which are often deregulated in cancer cells, establishing a link between signal transduction, alternative splicing, and gene expression during tumorigenesis [22, 32, 33] (Figure 2).

Sam68 is overexpressed in breast, prostate, renal, and cervical cancer cells [26, 34–36] and is also frequently upregulated in tumors [34, 37].

The first hard evidence that Sam68 is involved in regulation of alternative splicing with an impact on tumorigenesis was provided by the demonstration that it promotes inclusion of exon v5 in the CD44 pre-mRNA [33]. CD44 encodes a cell surface molecule involved in cancer cell proliferation. CD44 transcript isoforms are alternatively generated by the inclusion of 10 variant exons, which are decisive in tumor progression [38]. Depletion of Sam68 strongly reduces the inclusion of several variable exons. Interestingly, Sam68 activity is controlled by the Ras signaling pathway, and Sam68 phosphorylation by ERK is needed to promote v5 inclusion [33].

Sam68 also regulates AS of cyclin D1, a protooncogene frequently deregulated in cancer cells [39, 40]. In addition, Sam68 promotes the generation of a stable SF2/ASF, isoform through regulation of splicing. The protooncogene SF2/ASF, also a splicing factor, is in turn responsible for processing of ΔRon pre-mRNA, which encodes a factor involved in EMT in colon cancer cells [41].

Another connection of Sam68 with cancer could be provided by the control of AS of the Bcl-x transcript. The Bcl-x gene can yield the antiapoptotic Bcl-x(L) factor or the proapoptotic Bcl-x(S) [22, 42]. Some studies have reported that Sam68 overexpression causes the accumulation of proapoptotic Bcl-x(s) in a manner that depends on the RNA-binding activity of Sam68 [22, 42]. However, the observation that Sam68 and the antiapoptotic Bcl-x(L) are upregulated in prostate cancer cells is at odds with a proposed activity of Sam68 in Bclx(S) upregulation [34, 43]. This apparent contradiction was resolved by the finding that the activity of Sam68 on Bcl-x AS depends on its phosphorylation status, which can switch Sam68 function from proapototic to antiapoptotic in cancer cells. Indeed, Src-like kinase, which is often activated in cancer, phosphorylates Sam68 and thereby promotes splicing of the antiapoptotic Bcl-x(l) variant which inhibits cell death [22].

Intriguingly, in advanced breast and renal tumors, Sam68 was found to localize in the cytoplasm [26, 35]. These observations suggest a potential function of Sam68 in translational control in advanced stages of tumorigenesis. In accordance with a potential role of Sam68 in translation, it was previously proposed to regulate the translation of selected mRNAs in male germ cells and neurons [44, 45].

Other RBPs regulating splicing in cancer cells are hnRNPs (A/B) H, SR proteins (ASF/SF2), RBM5, HuR, and PTB. The interested reader can refer to the following reviews and articles [30, 46].

### 2.2. eIF4E Overexpression in Cancer Enhances Translation Initiation of Specific mRNAs

Translation initiation is a critical step of protein synthesis and is highly regulated [47]. One of the most crucial regulators is the cap-binding protein eIF4E (eukaryotic initiation factor 4E) [48]. In the cytoplasm, eIF4E binds directly to the m^7^GTP-cap structure present at the 5′end of all mRNAs and interacts with eIF4G, which in turn recruits the 43S ribosomal complex during initiation of translation. eIF4E and eIF4G together with the RNA helicase eIF4A form the eIF4F complex, which is often targeted for translational regulation [47].

Early findings indicated that eIF4E overexpression leads to malignant transformation of fibroblasts [49, 50]. Since then, numerous studies have reported overexpression of eIF4E in different tumor types (e.g., breast, prostate, gastric colon, lung, skin, and lymphomas) [51]. Elevated expression of eIF4E often correlates with malignancy and poor prognosis [52, 53]. Surprisingly, overexpression of eIF4E does not induce a global increase in protein synthesis but augments translation of a subset of mRNAs encoding mostly prooncogenic proteins [2, 54] (Figure 2). mRNAs regulated by eIF4E overexpression include those encoding components of the cell cycle machinery (cyclin D1, CDK2, c-myc, RNR2, ODC, surviving, Mcl-1, Bcl-2) or factors implicated in angiogenesis (VEGF, FGF-2, PDGF) and invasion (MMP9) [2, 51, 55, 56].

It has been proposed that mRNAs coding for proteins upregulated in oncogenesis contain long and highly structured 5′UTRs [10]. mRNAs bearing stable secondary structures in the 5′UTR are poorly translated in normal conditions and may be particularly dependent on the eIF4F complex and the unwinding capacity of the eIF4A helicase to initiate translation. Thus eIF4E overexpression may lead to enhanced translation of otherwise inefficiently translated transcripts involved in tumorigenesis [2]. Interestingly, eIF4E seems to be implicated in nucleocytoplasmic transport of mRNAs (e.g., cyclin D) and thus may regulate expression of some genes in an initiation-independent way [57].

eIF4E activity is regulated by signaling pathways amplified in human cancers (Figure 2). The protein kinase mTor phosphorylates eIF4E-binding proteins (4E-BP). In their unphosphorylated state, 4E-BPs bind to eIF4E on the same site recognized by eIF4G, blocking the formation of the cap-binding complex. Phosphorylation of 4E-BP leads to loss of affinity for eIF4E and increases translation [2]. In addition, eIF4E phosphorylation by MAPK-integrating kinases MNK1 and MNK2 enhances cap-dependent initiation [47, 54].

Given the important role of eIF4E in tumorigenesis, reducing either eIF4E activity or levels in cancer cells has become an attractive anticancer strategy [51, 58]. Many compounds inhibiting mTor kinase activity have proven to be efficient. For example, PP242, Tonin1, and INK128 are ATP active site inhibitors of mTOR and block the phosphorylation of all mTor targets including 4E-BP [51]. Unfortunately, cells of some cancer types are insensitive to treatment with mTor inhibitors [59]. As an alternative strategy, inhibiting eIF4E expression with antisense oligonucleotides (AON) has given promising results in suppressing tumor growth in vivo [60].

### 2.3. La Is an ITAF Implicated in Cancer

The multifunctional RNA-binding protein La is primarily nuclear but can shuttle between the nucleus and the cytoplasm [61, 62]. According to its localization, La functions in small RNA processing [63] and in translation of mRNAs [64–66]. La can be divided into three regions: the N-terminus, which contains the conserved La motif; a less conserved RNA recognition motif (RRM); and a weakly conserved C terminus, which contains an RRM, and a nuclear localization signal (NLS) [67] (Figure 1). The La motif folds into an RRM and its high conservation suggests that it carries out a specific function [68, 69]. La interacts with cellular and viral mRNAs and regulates IRES and cap-dependent translation initiation [64, 66, 70–73]. An IRES is a nucleotide sequence folding in a specific secondary structure that recruits ribosomes independently of the cap structure [74]. During cellular stress, cap-dependent translation is downregulated, and IRES-dependent translation of many mRNAs is favored [75]. For example, under the hypoxic conditions usually found in the interior of a tumor, IRES-mediated translation of the angiogenic factor VEGF is favored leading to vascularization of the tumor [76]. Specific RNA-binding proteins termed IRES transacting factors (ITAFS) are required to regulate IRES-dependent translation in cancer development [74]. La is an ITAF that regulates the IRES-dependent translation of mRNAs involved in cell proliferation, angiogenesis and apoptosis [64, 77, 78] (Figure 2).

As an ITAF, La interacts directly with the IRES of the mRNA encoding the proapoptotic factor XIAP [64]. In addition, La regulates IRES-dependent translation of LamB1, a factor that drives invasion, angiogenesis and metastasis [79, 80]. La also binds to the IRES of cyclin D1 (CCND1) in cervical cancer tissues, and its overexpression correlates with upregulation of cyclin D1 while its depletion leads to a reduction of cyclin D1 levels and a defect in cell proliferation [77].

La is overexpressed in chronic myeloid leukemia, cervical cancer tissues, oral squamous cell carcinoma (SCC), and in a number of cancer cell lines compared to nontumorigenic cells [66, 77, 78, 81]. In SCC, La is required for expression of *β*-catenin and MMP-2, proteins implicated in cell-cell adhesion and cell motility, respectively [78]. In leukemia, increased levels of La correlate with upregulation of MDM2 (an oncogenic tyrosine kinase). La interacts directly with the 5′UTR of mdm2 mRNA and enhances its translation [66].

Using mouse glial progenitor cells, Brennet proposed that La functions as a translational regulator during KRas/Akt oncogenic signaling [62]. Ras and Akt pathways are aberrantly active in cancer cells and play a pivotal role in the formation and regulation of glioblastoma [82]. In this tumor type La is phosphorylated by Akt, and this changes its distribution from the nucleus to the cytoplasm leading to association of a subset of La-bound mRNAs to polysomes. Many of these mRNAs encode factors implicated in oncogenesis such as Cyclin G2, Bcl2, and PDGFA [62].

The number of known La mRNA targets is still limited and further studies are necessary to understand its function in tumorigenesis. However, La already represents a promising target for cancer therapy. As an example, La activity has been efficiently blocked by a synthetic peptide corresponding to amino acids 11 to 28 of La. By competition, the peptide inhibits IRES-driven translation of Hepatitis C without affecting cap-dependent translation of cellular mRNAs [83]. This peptide could also be used to block expression of cancer related mRNA targets of La.

Other ITAFs implicated in cancer are PTB, hnRNP A1, hnRNP E1, hnRNP E2, and YB1. The interested reader can refer to the following reviews and articles [84, 85].

### 2.4. HuR Regulates the Stability and Translation of Cancer-Related Transcripts

The human antigen R (HuR) is the most prominent RBP known to be implicated in tumorigenesis [3]. Overexpression of HuR has been observed in lymphomas, gastric, breast, pancreatic, prostate, oral, colon, skin, lung, ovarian, and brain cancers [86–91]. Elevated cytoplasmic accumulation of HuR correlates with high-grade malignancy and serves as a prognostic factor of poor clinical outcome in some cancer types [92–95]. Localized in the nucleus of normal cells, HuR often translocates to the cytoplasm in transformed cells [96, 97]. HuR's subcellular localization is regulated by posttranslational modifications, and the enzymes modifying HuR are all implicated in cancer [97] (Figure 2). In the cytoplasm, HuR binds to adenine- and uridine-rich elements (AU-rich elements or AREs) located in 3′UTR of target mRNAs [98]. AU-rich elements serve as binding sites for a variety of RBPs that modulate mRNA half-life [11]. An estimated 10% of all mRNAs bear AU-rich sequences [99]. The minimal functional ARE sequence is a nonamer UUAUUUAWW [100]. Most RBPs binding to AREs promote rapid deadenylation and degradation of substrate mRNAs by targeting them to the exosome (e.g., TTP, AUF1, CUGBP2) [101]. On the contrary, HuR most often enhances the stability of its target mRNAs [3]. In addition, HuR can also regulate the splicing of a certain number of targets [102].

HuR is a member of the embryonic lethal abnormal vision (ELAV) family of proteins and contains three RRMs that provide high-affinity RNA binding [103] (Figure 1). HuR target mRNAs encode products that promote proliferation, inhibit apoptosis, increase angiogenesis, and facilitate invasion and metastasis. For an extensive list of HuR targets, see [3]. Below I will give an overview of HuR targets and will summarize the different mechanisms by which HuR regulates their expression.

Upon binding to the 3′UTR, HuR stabilizes the mRNAs coding for cyclins (cyclin D1, E1, A2, B1), favoring cell cycle progression and promoting proliferation of cancer cells [104–106]. HuR also promotes cancer cell survival by stabilizing transcripts encoding antiapoptotic factors like Bcl-2, Mcl-1, SIRT1, and p21 [90, 107–110]. mRNAs coding for proteins implicated in invasion and metastasis (MMP-9) [111, 112], cell migration and adhesion (Urokinase A and uPA receptor) [113] or EMT (snail) are also stabilized by HuR [114]. Expression of the proangiogenic factors VEGF and HIF-1*α* is controlled by HuR. Regulation of HIF-1*α* mRNA is interesting, as HuR binds to both the 5′ and 3′UTRs and promotes translation and stability [115, 116]. The mechanism by which HuR stabilizes its targets is still unclear, but recent studies have proposed an interplay between HuR and miRNAs [117]. HuR is able to suppress activity of miRNAs, by inhibiting their recruitment to the mRNA or even by promoting their downregulation. Some examples of cross-talk between HuR and miRNAs will be given in the next paragraph.

ERBB-2 overexpression is associated with development and progression of prostate cancer. HuR enhances ERBB-2 expression using a miRNA-dependent mechanism. HuR binds to a uridinerich element (URE) in the 3′UTR of ERBB-2 and inhibits action of miR-331-3p to a nearby site [118]. The presence of HuR on the mRNA does not alter miR-331-3p binding, which leads to the hypothesis that HuR may rather reduce association between ERBB-2 mRNA and the RNA silencing complex [118]. In colorectal cancer, HuR overexpression and localization in the cytoplasm correlate with decreased levels of miR-16, a miRNA that binds to the 3′UTR of COX-2 mRNA and inhibits its expression by mRNA decay [119]. Intriguingly, HuR interacts with miR-16 and promotes its downregulation in an mRNA ligand-dependent manner. Thus, HuR stabilizes COX-2 mRNA by binding to the ARE and by downregulating miR-16 [119].

Interestingly, HuR is able to repress the translation of the proapoptotic factor c-Myc by recruiting the let-7 miRNP to the 3′UTR [120]. HuR is not the only RBP which assists in targeting miRNPs to the 3′UTR of mRNAs, as was shown with the example of TTP [121].

HuR also represses the translation of some of its targets by binding to the 5′UTR. This is the case for p27, which prevents cell proliferation [122].

It has been recently shown that HuR can act as an ITAF binding to the IRES of XIAP mRNA, which encodes an anti-apoptotic factor [123]. HuR stimulates the translation of XIAP mRNA by binding to XIAP IRES and enhancing its recruitment into polysomes.

Interestingly in the case of the antiapoptotic factor prothymosin alpha (ProT*α*), HuR binding to its 3′UTR enhances nuclear export of the mRNA followed by induced translation upon UV irradiation [124].

In summary, the majority of HuR mRNA targets are stabilized upon binding, and translation is enhanced. As an ITAF, HuR binds to IRES structures and enhances translation. HuR is also able to inhibit translation by binding to 5′UTR or by recruiting miRNPs to the 3′UTR. On the other hand, HuR also inhibits miRNA binding to the 3′UTR of its target mRNAs. Finally, HuR is increasing cytoplasmic abundance of target mRNAs probably via enhanced mRNA nulear export. These examples illustrate the complexity of HuR regulatory activity.

The large spectrum of mRNA targets regulated by HuR confirms its potential to coordinate nearly all steps of tumorigenesis. Overexpressed in a high number of cancer types, HuR provides a good candidate for therapy design. Surprisingly, however, a recent study showed that elevated levels of HuR may be advantageous for cancer therapy. In pancreatic ductal adenocarcinoma, HuR levels modulate the therapeutic activity of gemcitabine (GEM), a common chemotherapeutic agent [125]. GEM exposure to cancer cells increases the amount of cytoplasmic HuR and promotes its association with dCK mRNA, which encodes the enzyme that activates GEM, establishing a positive feedback loop that improves its therapeutic efficacy. This example shows that therapies that reduce the level of HuR have to be designed carefully, and perhaps in a tumor type-dependent manner [126].

Besides HuR, a number of other factors can regulate the stability and expression of mRNAs bearing AREs [127]. The TIS11 family of RBPs composed of Tristetraprolin (TTP) and butyrate response factors 1 and 2 (BRF-1 and-2) bind and target ARE-containing mRNAs for rapid degradation [101]. AUF1 is able to stabilize or destabilize ARE-containing mRNAs [128]. The CELF family of RNA-binding proteins is composed of 6 members, which promote either mRNA decay or translation of its target mRNAs [129, 130]. For example CUGBP2 binds COX-2 mRNA which is then stabilized but translationally repressed [131]. T-cell intracellular antigen-1 (TIA-1) and TIA-1-related (TIAR) proteins are translational silencers [132]. Some of these factors have common targets and compete for binding depending on cellular conditions.

## 3. Conservation of RBPs across Eukaryotes

Post-transcriptional gene regulation is a coordinated, efficient, rapid and flexible mechanism to control the proteome of the cell in response to different physiological conditions. It is thus not surprising that some organisms have become highly dependent on post-transcriptional mechanisms to regulate gene expression, like, for example, the protozoan parasite, trypanosome [133–135]. The trypanosome genome encodes very few potential regulatory transcription factors, and gene regulation relies mostly on RNA-binding proteins [136]. It has been proposed that 3–11% of the proteome in bacteria, archea and eukaryotes are putative RNA-binding proteins [137]. The large number of RBPs suggests that RNA metabolism may be a central and evolutionarily conserved contributor to cell physiology. Most of the RNA-binding domains known today are present in early stages of evolution. Interestingly, several new eukaryotic-specific RNA-binding domains have emerged, like the RRM, which suggests that post-transcriptional gene regulation became more complex with evolution [137].

**Figure 3:**
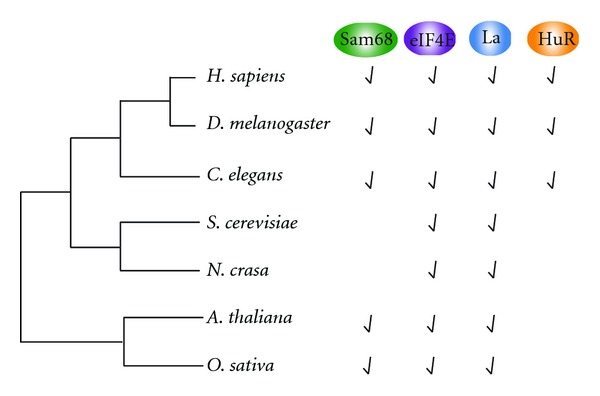
Conservation of Sam68, eIF4E, La, and HuR in different phyla. Phylogenetic tree of the RBPs described in this paper. The presence of homologues is indicated.

The RNA-binding proteins described in this paper are widely conserved across eukaryotes (Figure 3). We could detect homologues of HuR only in metazoa and not in fungi and plants. Human HuR is the most divergent family member of the ELAV proteins. While the other members, HuD, HuC, and Hel-N1, present a neuron- and brain-specific expression, where they are mostly implicated in alternative splicing, HuR is ubiquitously expressed and fulfills numerous functions [138, 139].

Sam68 homologues exist in all eukaryotes except fungi (Figure 3). In the STAR protein family, the Sam68 subfamily is composed of Sam68 (SRC-associated in mitosis, 68 kd) and the Sam68-like mammalian proteins 1 and 2 (SLM-1 and SLM-2, also named T-STAR in humans) [140–143]. As in the case of HuR, Sam68 is ubiquitously expressed, whereas SLM-1 and SLM-2 expression is restricted to few cell types or tissues [144]. In humans, Sam68 has acquired a larger spectrum of functions and plays a major role in signaling and splicing in different tissues.

Contrary to HuR and Sam68, La homologues can be identified in all three phyla: metazoa, fungi, and plants (Figure 3). La was first characterized as a human protein, and homologues have been identified in a wide variety of other eukaryotes [63]. The N-terminal part containing the La motif is highly conserved, in contrast to the C-terminal domain which varies both in size and sequence between species, ranging from 70 amino acids in the yeasts *S. cerevisiae *and *S. pombe *to more than 220 amino acids in vertebrates. Human La is phosphorylated at different sites, all located in the C terminus [63] (Figure 2). Interestingly these sites are only conserved in vertebrate La proteins. The presence of an additional C-terminal region including different functional domains and phosphorylation sites shows that La has evolved to a highly regulated and multifunctional factor in vertebrates.

The translation initiation factor eIF4E is highly conserved across eukaryotes. Sequence comparisons revealed a phylogenetically conserved 182 amino acid C-terminal region [145, 146]. In contrast, the N-terminal region is poorly conserved and is not required for cap-dependent translation [145]. Functional conservation has also been demonstrated, as mammalian eIF4E can rescue the lethality caused by disruption of the yeast eIF4E gene [147]. The crystallographic structure of eIF4E in mouse, yeast, human, and wheat has been solved [145, 148–150]. The three-dimensional structure of the C-terminal part of murine eIF4E demonstrates that the surface of the molecule resembles a cupped hand that contains a narrow cap-binding slot. The remarkable level of sequence identity across phylogeny suggests that all known eIF4Es share the same structure in their conserved C-terminal region [145]. eIF4E thus does not contain a canonical RBD but adopts a conserved three-dimensional structure which interacts with the cap.

Interestingly most eukaryotic organisms express multiple eIF4E family members, and it has been proposed that a ubiquitously expressed member of the family may be implicated in general translation initiation while others could be involved in specialized functions [151, 152]. eIF4E family members may provide an additional layer of control in translation and may regulate specific subsets of mRNAs, which could be linked to cancer development.

## 4. Concluding Remarks

In cancer research, the impact of post-transcriptional gene regulation has been considered only since a few years. Today, it is well established that a subset of RBPs are key regulators of processes involved in tumorigenesis. The genome wide analysis of RBPs and their RNA targets has allowed a better understanding of the complex world of mRNA metabolism and the connections existing between different RBPs. According to the “RNA operon” concept, mRNAs encoding functionally related proteins are coregulated by specific RBPs, ensuring an efficient, flexible, and coordinated response to cellular need [144, 153, 154]. RNA operons can be interconnected. HuR and eIF4E for example, share common mRNA targets like c-myc, cyclin D1 and VEGF, suggesting an orchestrated regulation of the expression of genes implicated in tumorigenesis [155, 156]. In addition, HuR regulates expression of eIF4E in cancer cells [156]. These observations show that post-transcriptional regulation events are highly linked and provide a powerful mechanism to control the fate of a cell.

RBPs are highly versatile factors that can bind to multiple RNA targets and regulate their fate by a variety of mechanisms. The fact that every step of the mRNA life cycle is narrowly controlled allows RBPs to fine tune expression in a very precise manner. The conservation of RBPs across eukaryotes and the emergence of more complexity along evolution also point to an essential role of RBPs. Post-transcriptional gene regulation is a central mechanism of emerging importance in cancer research which is expected to provide novel targets for therapy design.
